# Supplementary material for: The cofactor-dependent folding mechanism of Drosophila cryptochrome revealed by single-molecule pulling experiments
Source: Nat Commun. 2023 Feb 24;14:1057. doi: 10.1038/s41467-023-36701-y (PMC9958137; doi:10.1038/s41467-023-36701-y)
Supplement: Supplementary file 1 — Supplementary Information [file 41467_2023_36701_MOESM1_ESM.pdf]

SUPPLEMENTARY INFORMATION

to

The cofactor-dependent folding mechanism of *Drosophila* cryptochrome revealed by single-molecule pulling experiments

Sahar Foroutannejad[1], Lydia L. Good[1], Changfan Lin[2], Zachariah I. Carter[3], Mahlet G. Tadesse[4], Aaron L. Lucius[3], Brian R. Crane[2], Rodrigo A. Maillard[1,#]

[1] Department of Chemistry, Georgetown University, Washington, DC, USA
[2] Department of Chemistry & Chemical Biology, Cornell University, Ithaca, NY, USA
[3] Department of Chemistry, University of Alabama at Birmingham, Birmingham, AL, USA
[4] Department of Mathematics and Statistics, Georgetown University, Washington, DC, USA

# Corresponding author: Rodrigo A. Maillard (rodrigo.maillard@georgetown.edu)

This document contains the following:

- Supplementary Figure 1: Modification of dCRY for single-molecule optical tweezers experiments
- Supplementary Figure 2: Spectroscopic characterization of wild type and tagged dCRY
- Supplementary Figure 3: Time-dependent histograms of degree of folding of dCRY
- Supplementary Figure 4: Model-based clustering of single-molecule data
- Supplementary Figure 5: Histograms of population of dCRY degree of folding in the presence of different cofactors
- Supplementary Figure 6: Identification of intermediate structures based on clusters centers
- Supplementary Figure 7: Average first-passage time from the unfolded to the native state
- Supplementary Table 1: Clustering analysis of degree of folding data
- Supplementary Table 2: Fitted parameters of kinetics refolding models
- Supplementary Table 3: Statistical analysis of kinetic refolding models
- Supplementary Table 4: Degree of folding of dCRY in the presence of FAD moieties
- Supplementary Table 5: Analysis of total change in contour length of each dCRY cluster

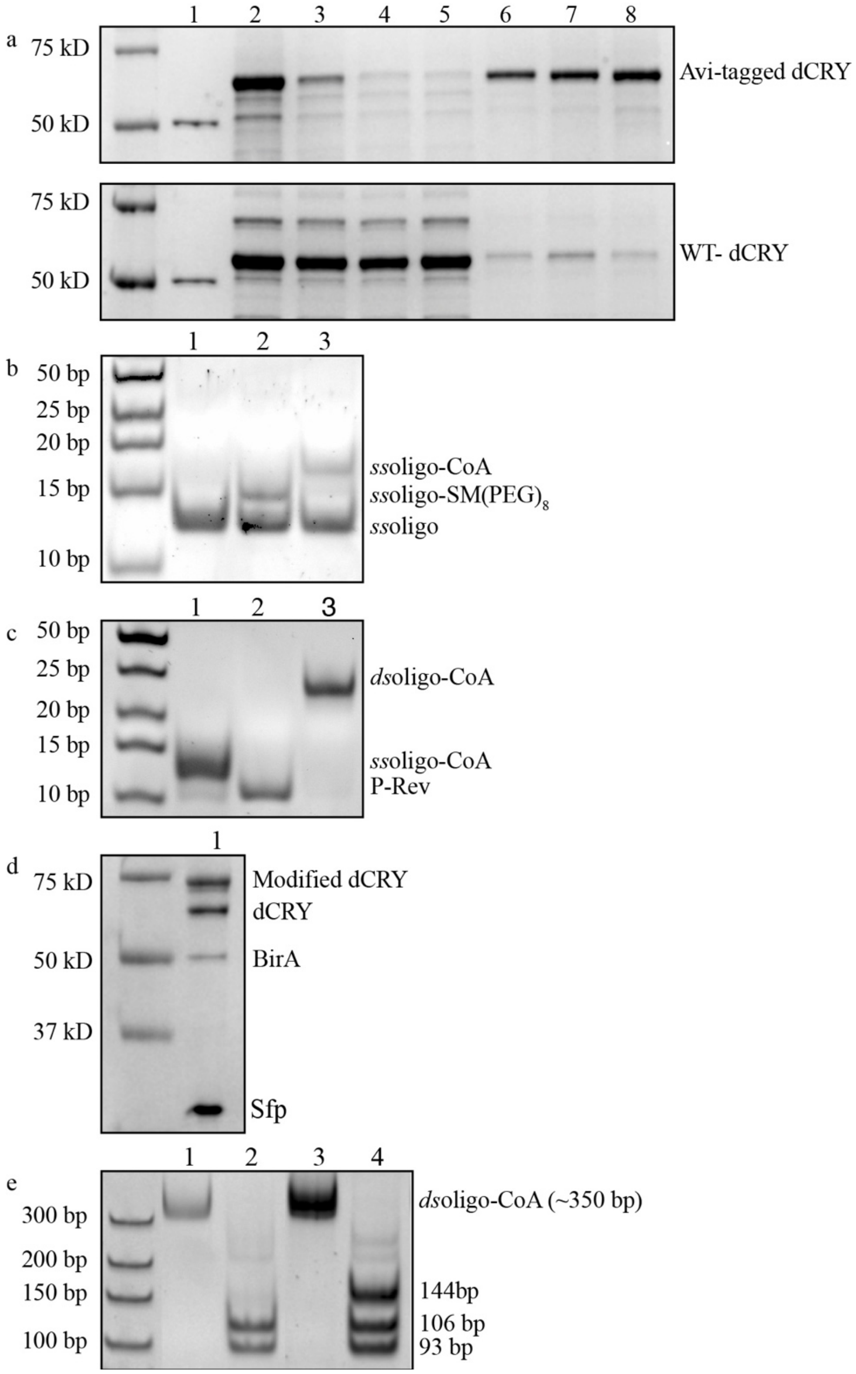

Supplementary Figure 1. Modification of dCRY for single-molecule optical tweezers experiments. a- Top panel is the biotinylation reaction of tagged dCRY with Avi-tag and the bottom panel is WT-dCRY as control. Lane 1: BirA; Lane 2: tagged dCRY (top) and WT-dCRY (bottom); Lanes 3-5: supernatant collected from 30, 60 and 90 ul; Lanes 6-8: beads resuspended in PBS and loaded into the gel. 10% SDS-PAGE, 35 mA, 50 minutes. Gels were stained in Coomassie blue and distained with 10% acetic acid and 20 % methanol in distilled water. b- Amine to sulfhydryl modification of single stranded oligonucleotide (ssOligo) with acetyl-CoA via a

bifunctional $(PEG)_8$ linker. Lane 1: ssOligo; Lane 2: modification of ssOligo with PEG linker; Lane 3: final product (ssOligo-CoA). 15% PAGE, 100 V, 150 minutes, stained with ethidium bromide solution. c- Annealing reaction: Lane 1: ssOligo-CoA; Lane 2: phosphorylated complementary sequence (P-rev); Lane 3: double stranded modified oligo nucleotide (dsOligo-CoA). 15% PAGE, 100 V, 150 minutes, stained with ethidium bromide solution. d- Modification of dCRY by dsOligo-CoA mediated by the enzyme Sfp. e- Ligation of 315-bp DNA handle to the dsOligo of the modified dCRY. The DNA handle has restriction sites for the restriction enzyme PvuII. After productive ligation to the dsOligo-CoA from dCRY and digestion, it results in three DNA fragments of 93, 106 and 144 bps. All experiments shown in panels a-e where repeated at least 3 times, yielding similar results.

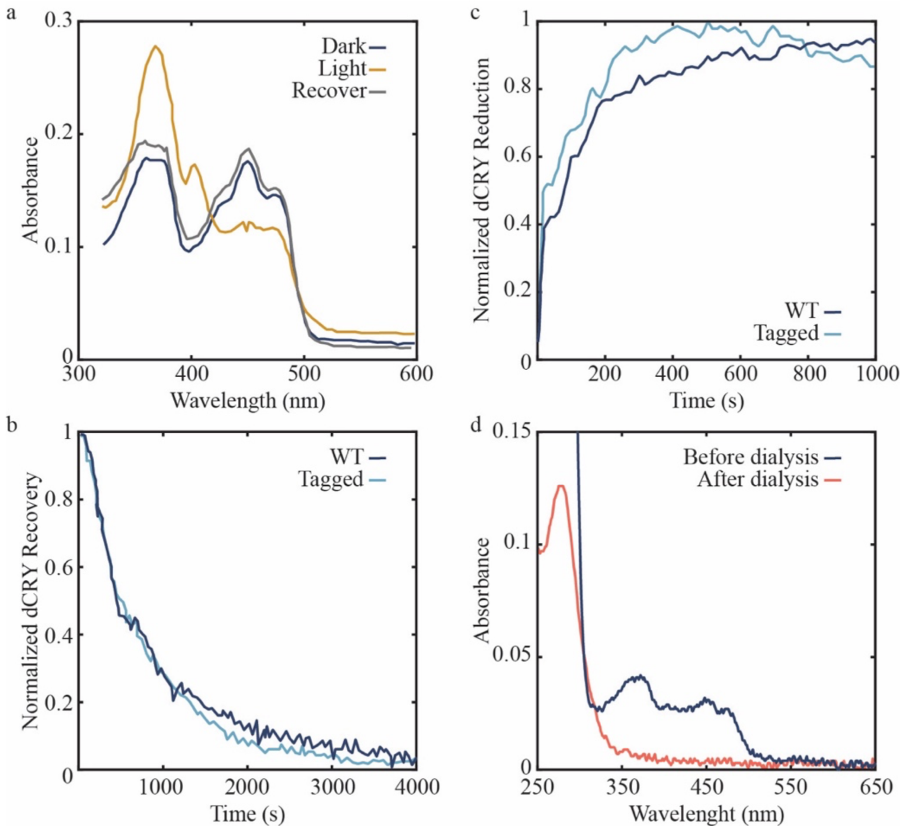

Supplementary Figure 2. Spectroscopic characterization of wild type and tagged dCRY. a- dCRY modified with Avi and ybbR tags does not affect redox state response of FAD to light (active), dark (inactive), and recovery after activation with light (Recover). b-c- kinetics of reduction and recovery are the same between wild type dCRY and the tagged version used in this study. d- dCRY-FAD absorption spectra before and after dialysis in dCRY buffer (Methods).

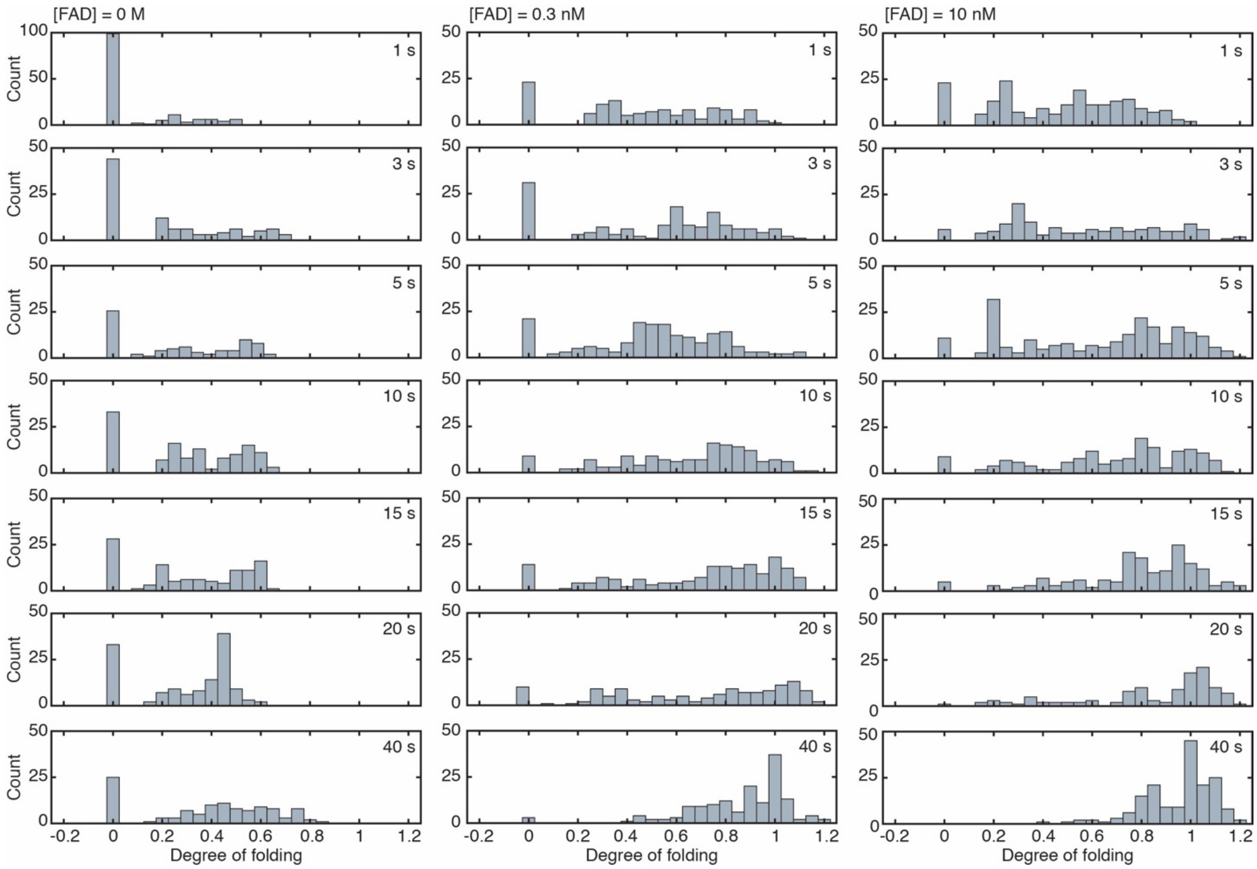

Supplementary Figure 3. Time-dependent histograms of degree of folding of dCRY. The histograms correspond to three FAD concentrations of 0 (left), 0.3 nM (middle) and 10 nM (right). Source data is available as a Source Data file.

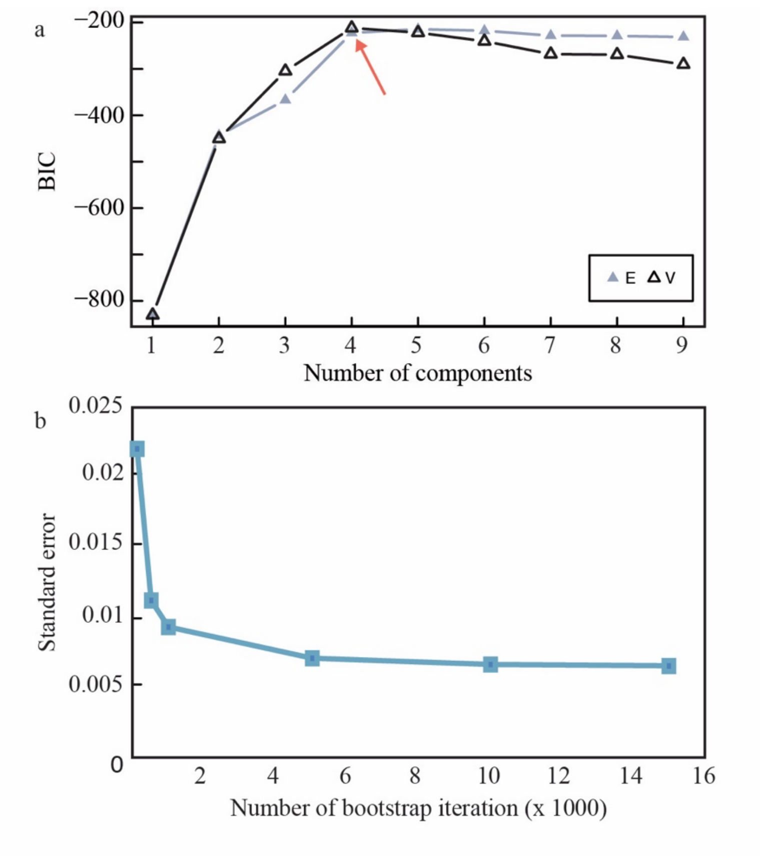

Supplementary Figure 4. Model-based clustering of single-molecule data. a- The red arrow shows the number of clusters favored by BIC when considering the data without the "zero values" that represent the unfolded state. Four clusters are selected for both mixture models considered: one assumed equal or same variance across clusters and is denoted "E", the other assumed varying or different variance parameters across clusters and is denoted "V". b- Standard error of one of the cluster centers. The standard errors for all model parameters were found to level off by 10000 bootstrap replications. Because the data have been standardized (observed total change in contour divided by the value expected for the natively folded protein) the cluster means and their standard errors have no units.

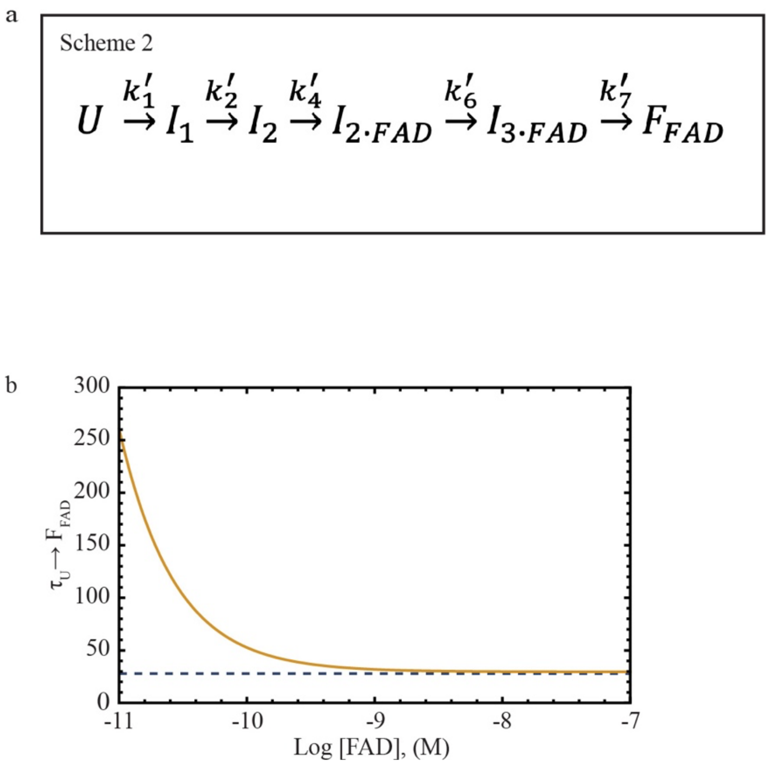

Supplementary Figure 5. Average first-passage time from the unfolded to the native state. a- Scheme 2 to determine the average time dCRY takes to fold into the native, FAD bound state starting from the unfolded state. The net rate constants from the unfolded to the folded state in Scheme 2 are obtained from the original rate constants in Scheme 1 (Figure 3g) multiplied by the partition between forward and backward flux (Methods). b- The plot shows the average time is takes from the unfolded state to reach the folded, FAD bound state (beige, solid line). The calculations are shown in Methods (Equations 4-9). The average time reaches a plateau of 30 s when [FAD] >> Kd (blue, dashed line). The cumulative probability distribution of an exponential function using a time constant of 30 s indicates that the probability of folding at 40 s is 0.73, a value that is consistent with the observed folded fraction of 0.66 +/- 0.4 (Figure 3i).

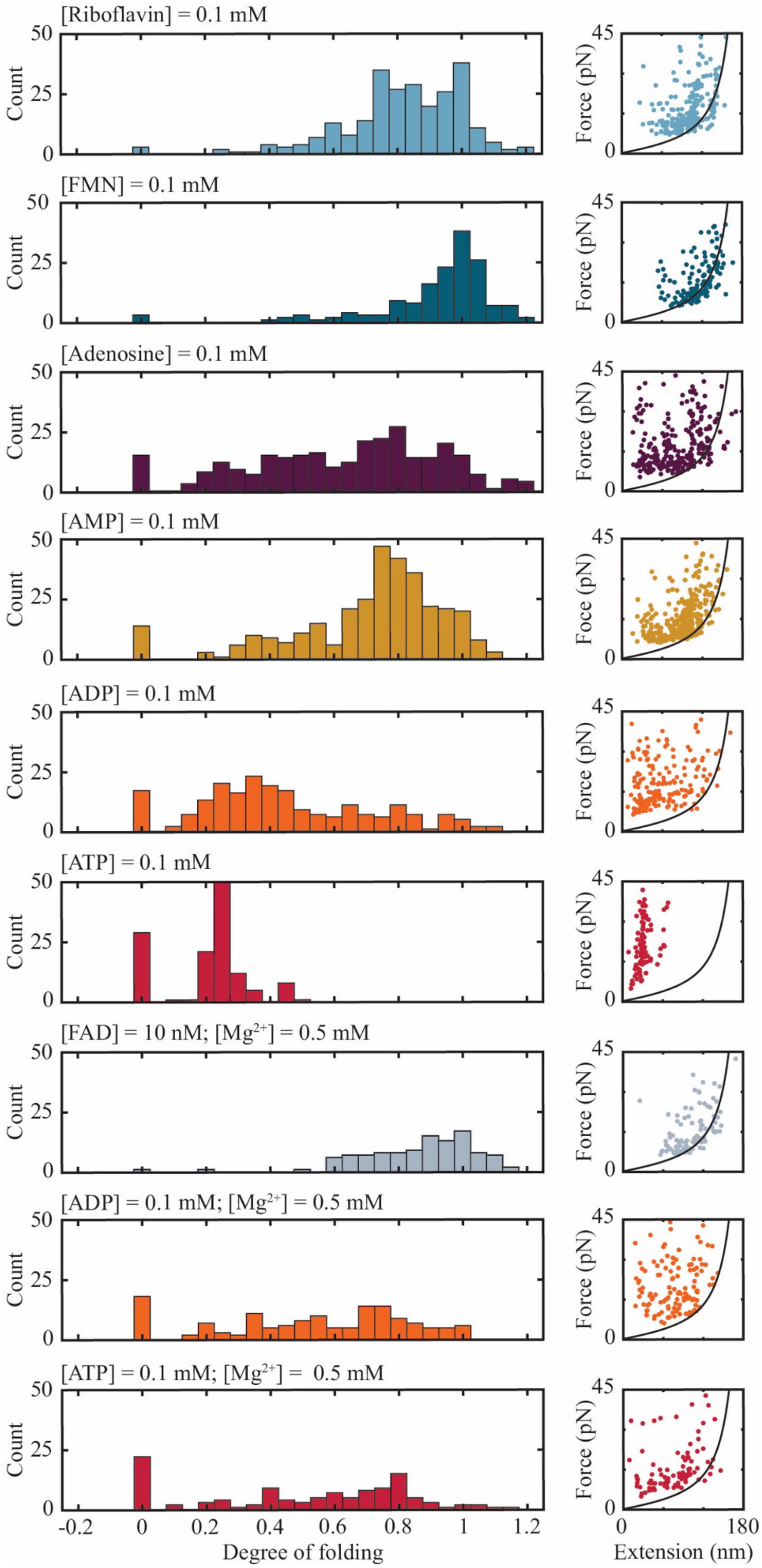
[Riboflavin] = 0.1 mM
[FMN] = 0.1 mM
[Adenosine] = 0.1 mM
[AMP] = 0.1 mM
[ADP] = 0.1 mM
[ATP] = 0.1 mM
[FAD] = 10 nM; [Mg2+] = 0.5 mM
[ADP] = 0.1 mM; [Mg2+] = 0.5 mM
[ATP] = 0.1 mM; [Mg2+] = 0.5 mM
Count
Force (pN)
Foce (pN)
Degree of folding
Extension (nm)

Supplementary Figure 6. Histograms of population of dCRY degree of folding in the presence of different cofactors. On the right of each histogram is the Worm-like chain analysis of each data point with the black line indicating the WLC for the fully folded dCRY. Source data is available as a Source Data file.

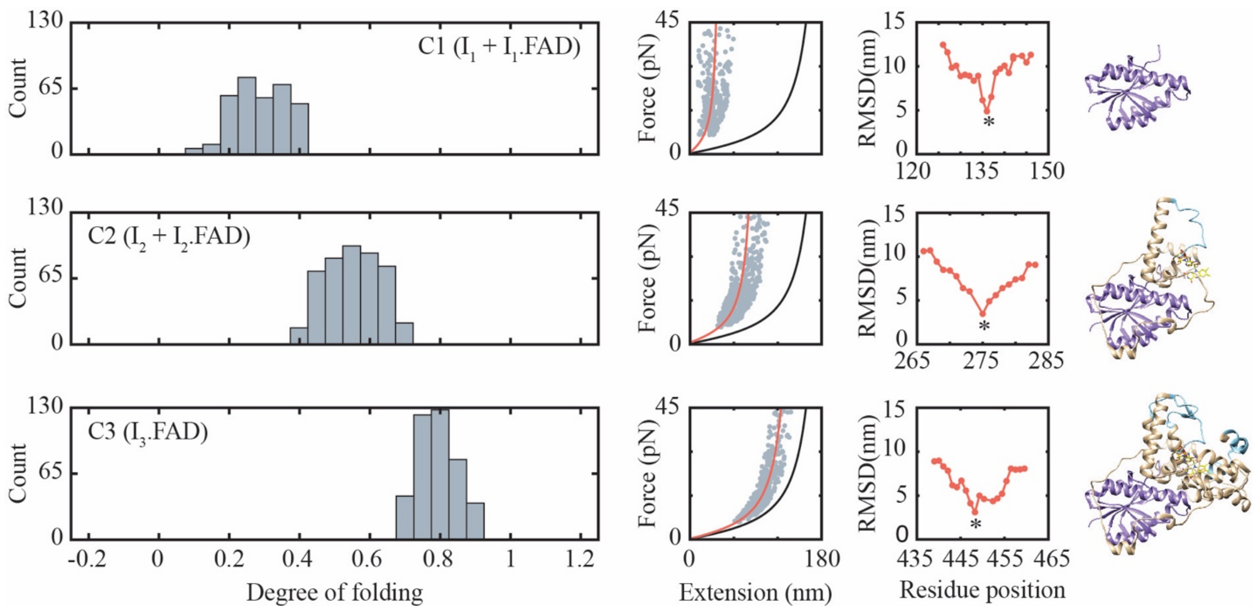

Supplementary Figure 7. Identification of intermediate structures based on clusters centers. Histograms of population of dCRY degree of folding in each cluster (left) and the WLC analysis of the data according to the changes in contour length in each cluster (middle). The lines in black and red correspond to the WLC model for the fully folded state and intermediates, respectively. Plot of absolute values of the root-mean-square-deviation (R.M.S.D.) between the observed data in each cluster and WLC models as a function of residue position shows approximate number of folded amino acids in each intermediate (right) (Methods). Source data is available as Source Data file. *Indicates the residue position with the lowest R.M.S.D.

Supplementary Table 1. Clustering analysis of degree of folding data*

**Refolding Kinetic Experiment at [FAD] = 0 M**						
		**C0**	**C1**	**C2**	**C3**	**C4**
**With Zero-valued Points**	**Mean**	0.0	0.27	0.47	--	--
	**Variance**	0.01	0.01	0.01	--	--
	**N**	805				
**Without Zero-valued Points**	**Mean**	0	0.22	0.43	--	--
	**Variance**	0	0.00	0.17	--	--
	**N**	805				
**Refolding Kinetic Experiment at [FAD] = 0.3 nM**						
**With Zero-valued Points**		**C0**	**C1**	**C2**	**C3**	**C4**
	**Mean**	0.01	0.32	0.54	0.78	1.00
	**Variance**	0.01	0.01	0.01	0.01	0.01
	**N**	1263				
**Without Zero-valued Points**	**Mean**	0	0.30	0.53	0.77	1.00
	**Variance**	0	0.01	0.01	0.01	0.01
	**N**	1263				
**Refolding Kinetic Experiment at [FAD] = 10 nM**						
**With Zero-valued Points**		**C0**	**C1**	**C2**	**C3**	**C4**
	**Mean**	--	0.27	0.58	0.79	1.01
	**Variance**	--	0.03	0.01	0.01	0.01
	**N**	1347				
**Without Zero-valued Points**	**Mean**	0	0.27	0.54	0.78	1.02
	**Variance**	0	0.01	0.01	0.01	0.01
	**N**	1347				

Supplementary Table 1. Clustering analysis of degree of folding data (continued)

Global Analysis of Refolding Kinetic Experiment of all FAD concentrations						
		**C0**	**C1**	**C2**	**C3**	**C4**
**With Zero-valued Points**	**Mean**	0.01	0.29	0.53	0.78	1.01
	**Variance**	0.001	0.004	0.005	0.005	0.0004
	**N**	3415				
**Without Zero-valued Points**	**Mean**	0	0.25	0.51	0.79	1.00
	**Variance**	0	0.01	0.01	0.008	0.008
	**N**	3415				
**FAD Titration**						
**With Zero-valued Points**	**Mean**	0.01	0.29	0.53	0.76	0.99
	**Variance**	0.005	0.005	0.005	0.555	0.005
	**N**	2139				
**Without Zero-valued Points**	**Mean**	0	0.28	0.50	0.76	0.99
	**Variance**	0	0.005	0.01	0.009	0.007
	**N**	2139				
**Combined FAD titration and Refolding Kinetic Experiment of all FAD concentrations**						
**With Zero-valued Points**	**Mean**	00.01	0.28	0.52	0.78	1.01
	**Variance**	0.005	0.005	0.006	0.005	0.005
	**N**	5554				
**Without Zero-valued Points**	**Mean**	0	0.26	0.52	0.79	1.02
	**Variance**	0	0.02	0.02	0.004	0.004
	**N**	5554				

* Source data is available as a Source Data file.

Supplementary Table 2. Fitted parameters of kinetics refolding models*

Model	k_1 (s^{-1})	k_{-1} (s^{-1})	k_2 (s^{-1})	k_{-2} (s^{-1})	k_3 ($M^{-1}\cdot s^{-1}$)	k_{-3} (s^{-1})	k_4 ($M^{-1}\cdot s^{-1}$)	k_{-4} (s^{-1})	k_5 (s^{-1})	k_{-5} (s^{-1})	k_6 (s^{-1})	k_{-6} (s^{-1})	k_7 (s^{-1})	k_{-7} (s^{-1})	χ^2
**Kinetic Refolding Data**															
FAD binding to I_1 and I_2	0.20 ± 0.04	0.17 ± 0.05	0.17 ± 0.03	0.06 ± 0.02	$2.8\cdot10^9$ ± $3\cdot10^8$	0.82 ± 0.08	$1\cdot10^{10}$ ± $1\cdot10^9$	1.49 ± 0.4	0.14 ± 0.03	0.03 ± 0.01	0.90 ± 0.10	0.46 ± 0.09	0.101 ± 0.008	0.026 ± 0.006	100
FAD binding to I_1 only	0.19 ± 0.04	0.18 ± 0.06	0.20 ± 0.03	0.058 ± 0.008	$1\cdot10^{10}$	1.3 ± 0.5	0	0	0.43 ± 0.05	2.2 ± 0.7	424 ± 1	3.1 ± 0.6	0.14 ± 0.02	0.06 ± 0.01	125
FAD binding to I_2 only	0.07 ± 0.01	0.01 ± 0.01	0.13 ± 0.02	0.03 ± 0.01	0	0	$1\cdot10^{10}$	1.4 ± 0.5	0	0	1.0 ± 0.2	0.5 ± 0.1	0.098 ± 0.009	0.024 ± 0.007	134
**FAD Titration**															
Model	k_1 (s^{-1})	k_{-1} (s^{-1})	k_2 (s^{-1})	k_{-2} (s^{-1})	k_3 ($M^{-1}\cdot s^{-1}$)	k_{-3} (s^{-1})	k_4 ($M^{-1}\cdot s^{-1}$)	k_{-4} (s^{-1})	k_5 (s^{-1})	k_{-5} (s^{-1})	k_6 (s^{-1})	k_{-6} (s^{-1})	k_7 (s^{-1})	k_{-7} (s^{-1})	χ^2
FAD binding to I_1 and I_2	0.65 ± 0.20	0.22 ± 0.05	0.40 ± 0.04	0.22 ± 0.02	$1\cdot10^{10}$ ± $1\cdot10^8$	5.1 ± 5.3	$5\cdot10^9$ ± $1\cdot10^7$	1.9 ± 0.4	0.31 ± 0.001	0.13 ± 0.5	0.49 ± 0.07	0.19 ± 0.02	0.93 ± 0.004	0.034 ± 0.001	78
FAD binding to I_1 only	0.7 ± 168	0.2 ± 0.8	0.0017 ± 0.0006	0.0010 ± 0.0003	$1\cdot10^{10}$	$1\cdot10^{10}$ ± 87000	0	0	0.2 ± ~0	18 ± ~0	29 ± ~0	0.2 ± ~0	1.3 ± ~0	0.001 ± ~0	$7.2\cdot10^3$
FAD binding to I_2 only	300 ± 40	100 ± 20	1.9 ± 0.2	1.2 ± 0.1	0	0	$1\cdot10^{10}$	4 ± 1	0	0	1.1 ± 0.2	0.43 ± 0.07	0.193 ± 0.009	0.073 ± 0.003	108

Supplementary Table 2. Fitted parameters of kinetics refolding models (continued)

**Global Analysis of Kinetic Refolding and FAD Titration Data**															
Model	k_1 (s^{-1})	k_{-1} (s^{-1})	k_2 (s^{-1})	k_{-2} (s^{-1})	k_3 ($M^{-1}\cdot s^{-1}$)	k_{-3} (s^{-1})	k_4 ($M^{-1}\cdot s^{-1}$)	k_{-4} (s^{-1})	k_5 (s^{-1})	k_{-5} (s^{-1})	k_6 (s^{-1})	k_{-6} (s^{-1})	k_7 (s^{-1})	k_{-7} (s^{-1})	χ^2
FAD binding to I_1 and I_2	0.15 ± 0. 03	0.09 ± 0.03	0.16 ± 0.03	0.06 ± 0.02	$2.8\cdot10^9$ ± $4\cdot10^8$	0.7 ± 0.3	$1\cdot10^{10}$ ± $9\cdot10^8$	2.9 ± 0.5	0.20 ± 0.04	0.09 ± 0.06	0.9 ± 0.1	0.38 ± 0.08	0.12 ± 0.04	0.044 ± 0.004	215
FAD binding to I_1 only	0.11 ± 0.04	0.04 ± 0.05	0.21 ± 0.05	0.12 ± 0.04	$1\cdot10^{10}$	$1\cdot10^6$ ± ~0	0	0	$1\cdot10^5$ ± ~0	0.001 ± 0.8	130 ± 20	6 ± 3	410 ± 20	360 ± 20	$7.7\cdot10^3$
FAD binding to I_2 only	0.13 ± 0.02	0.05 ± 0.02	0.18 ± 0.03	0.09 ± 0.02	0	0	$1\cdot10^{10}$	2.9 ± 0.5	0	0	1.0 ± 0.2	0.40 ± 0.09	0.12 ± 0.01	0.044 ± 0.005	279

* Source data is available as a Source Data file

Supplementary Table 3. Statistical analysis of kinetic refolding models*

Data Set[a]	Refolding Models[b]	N[c]	Fitted Para-meters[d]	Degrees of freedom[e]	$(\chi^2)^d$	Model Comparison			
						Model 2 vs. 1		Model 3 vs. 1	
						f_{obs}	p-value[f]	f_{obs}	p-value[f]
Kinetic data only	model 1	91	14	77	100	1.22	0.193	1.27	0.143
	model 2	91	12	79	125				
	model 3	91	10	81	134				
FAD Titration data only	model 1	50	14	36	78	87.4	0	1.25	0.253
	model 2	50	12	38	7200				
	model 3	50	10	40	108				
Kinetic & Titration data	model 1	141	14	127	215	35.3	0	1.26	0.097
	model 2	141	12	129	7700				
	model 3	141	10	131	279				

* We used the *F*-test statistic to determine the best fitting model to the kinetic and FAD titration data shown in Figure 3.

[a] Data set being used for the fitting of refolding models.

[b] In model 1, FAD binds to both intermediates, I_1 and I_2. In model 2, FAD only binds to I_1. In model 3, FAD only binds to I_2.

[c] Number of data points for each data set.

[d] Data obtained from Supplementary Table 2.

[e] Degrees of freedom (ν) is obtained by taking the difference between N and the number of fitted parameters.

[f] p-value evaluating the hypothesis that models 2 or 3 fit the data as well as model 1. There is strong evidence that model 1 provides a more robust fit than models 2 and 3 using the combined Kinetic and FAD Titration data sets.

Supplementary Table 4. Degree of folding of dCRY in the presence of FAD moieties*

		Percentage ± Standard Error				
**Moiety**	**Concentration**	**Folded**	**Intermediate (bound)[a]**	**Intermediate (unbound)[b]**	**Unfolded**	**N**
**Riboflavin**	0.1 mM	39 ± 0.1	61 ± 0.1	--	--	256
**FMN**	0.1 mM	84 ± 0.09	13.5 ± 0.2	--	2.5 ± 0.5	158
**Adenosine**	0.1 mM	20.3 ± 0.1	74.5 ± 0.07	--	5.2 ± 0.2	286
**AMP**	0.1 mM	23.5 ± 0.1	54.4 ± 0.07	17.8 ± 0.1	4.3 ± 0.3	327
**ADP**	0.1 mM	8.8 ± 0.2	25.6 ± 0.1	57.7 ± 0.1	7.9 ± 0.2	215
**ATP**	0.1 mM	0	0	77.3 ± 0.1	22.7 ± 0.1	128
**Addition of [Mg^{2+}] = 0.5 mM**						
**ADP**	0.1 mM	11.3 ± 0.2	49.3 ± 0.1	26.7 ± 0.2	12.7 ± 0.2	142
**ATP**	0.1 mM	11.4 ± 0.2	45.6 ± 0.1	23.7 ± 0.2	19.3 ± 0.2	114

[a] Intermediate bound states have a degree of folding between 0.5-0.8.
[b] Intermediate unbound states are considered when the observed degree of folding is less than 0.5.
* Source data is available as a Source Data file.

Supplementary Table 5. Analysis of total change in contour length of each dCRY cluster*

	**Nº of Residues**	Lc **(nm)**	ΔLc **(nm)**	x **(nm)**	**Residues in Sequence[a]**
**C1**	140	47.1	45.3	1.8	A6 to L135
**C2**	280	97	94.5	2.5	A6 to S276
**C3**	446	161	155.6	5.4	A6 to D448
**C4**	Full length	204	198.5	5.5	A6 to L516

[a] The number of residues of each intermediate, C1, C2 and C3 were obtained from the WLC analysis shown in Supplementary Figure 6.
* Source data is available as a Source Data file.
