# Supplementary material for: The cofactor-dependent folding mechanism of Drosophila cryptochrome revealed by single-molecule pulling experiments
Source: Nat Commun. 2023 Feb 24;14:1057. doi: 10.1038/s41467-023-36701-y (PMC9958137; doi:10.1038/s41467-023-36701-y)
Supplement: Supplementary file 2 — Reporting Summary [file 41467_2023_36701_MOESM2_ESM.pdf]

# nature portfolio

Corresponding author(s): Rodrigo A. Maillard

Last updated by author(s): Feb 2, 2023

# Reporting Summary

Nature Portfolio wishes to improve the reproducibility of the work that we publish. This form provides structure for consistency and transparency in reporting. For further information on Nature Portfolio policies, see our Editorial Policies and the Editorial Policy Checklist.

## Statistics

For all statistical analyses, confirm that the following items are present in the figure legend, table legend, main text, or Methods section.

| n/a | Confirmed | |
|---|---|---|
| ☐ | ☒ | The exact sample size ($n$) for each experimental group/condition, given as a discrete number and unit of measurement |
| ☐ | ☒ | A statement on whether measurements were taken from distinct samples or whether the same sample was measured repeatedly |
| ☐ | ☒ | The statistical test(s) used AND whether they are one- or two-sided<br>*Only common tests should be described solely by name; describe more complex techniques in the Methods section.* |
| ☒ | ☐ | A description of all covariates tested |
| ☒ | ☐ | A description of any assumptions or corrections, such as tests of normality and adjustment for multiple comparisons |
| ☐ | ☒ | A full description of the statistical parameters including central tendency (e.g. means) or other basic estimates (e.g. regression coefficient) AND variation (e.g. standard deviation) or associated estimates of uncertainty (e.g. confidence intervals) |
| ☐ | ☒ | For null hypothesis testing, the test statistic (e.g. $F$, $t$, $r$) with confidence intervals, effect sizes, degrees of freedom and $P$ value noted<br>*Give P values as exact values whenever suitable.* |
| ☒ | ☐ | For Bayesian analysis, information on the choice of priors and Markov chain Monte Carlo settings |
| ☒ | ☐ | For hierarchical and complex designs, identification of the appropriate level for tests and full reporting of outcomes |
| ☒ | ☐ | Estimates of effect sizes (e.g. Cohen's $d$, Pearson's $r$), indicating how they were calculated |

*Our web collection on statistics for biologists contains articles on many of the points above.*

## Software and code

Policy information about availability of computer code

| | |
|---|---|
| Data collection | We use a custom made labview based software to collect single molecule optical tweezers data. The software is available upon request or found here: http://tweezerslab.unipr.it/cgi-bin/software.pl/Search |
| Data analysis | We use Matlab and R based codes to do the following analyses: 1) Using Matlab we determine unfolding or refolding forces and the associated change in extension of all experiments with varying refolding times and various FAD concentrations. 2) We use the “mclust” package in R to perform model-based clustering analysis and of the FAD titration data and kinetic refolding data. We then used a bootstrap to estimate the uncertainty associated with the assignment of each data point into its designated cluster. Details of the procedures are in Methods section "Determining the Number of Clusters," "Bootstrap Analysis of  Clusters" and "Cluster Analysis of Cofactors Contained in FAD". 3) We use the freely available Matlab software MENOTR (Multi-start Evolutionary Nonlinear OpTimizeR) to fit the kinetic refolding data and FAD titration data to various models. The codes are available on on zenodo.org and are freely available (https://doi.org/10.5281/zenodo.7600414, https://doi.org/10.5281/zenodo.7600386). |

For manuscripts utilizing custom algorithms or software that are central to the research but not yet described in published literature, software must be made available to editors and reviewers. We strongly encourage code deposition in a community repository (e.g. GitHub). See the Nature Portfolio guidelines for submitting code & software for further information.

## Data

Policy information about availability of data

All manuscripts must include a data availability statement. This statement should provide the following information, where applicable:

- Accession codes, unique identifiers, or web links for publicly available datasets
- A description of any restrictions on data availability
- For clinical datasets or third party data, please ensure that the statement adheres to our policy

The data generated in this study, including all figures and tables, are provided in the Source Data file.

## Human research participants

Policy information about studies involving human research participants and Sex and Gender in Research.

| | |
|---|---|
| Reporting on sex and gender | Not applicable |
| Population characteristics | Not applicable |
| Recruitment | Not applicable |
| Ethics oversight | Not applicable |

Note that full information on the approval of the study protocol must also be provided in the manuscript.

# Field-specific reporting

Please select the one below that is the best fit for your research. If you are not sure, read the appropriate sections before making your selection.

[x] Life sciences [ ] Behavioural & social sciences [ ] Ecological, evolutionary & environmental sciences

For a reference copy of the document with all sections, see nature.com/documents/nr-reporting-summary-flat.pdf

# Life sciences study design

All studies must disclose on these points even when the disclosure is negative.

| | |
|---|---|
| Sample size | For each experimental condition, we collected data from a minimum of 6 different protein molecules and > 150 repetitions. Details of exact numbers of repetitions are in Supplementary Table 1. |
| Data exclusions | Single molecule unfolding or refolding data outputs force and changes in extension. We excluded data that showed more than +/- 2 standard deviation of the mean of changes in extension. These exclusion limits represented less than 1% of the total data collected. |
| Replication | For each experimental condition, we collected data from a minimum of 6 different protein molecules (biological replicates) |
| Randomization | This is not relevant to the study. Molecules corresponding to the same protein, dCRY, are subjected to optical tweezers under different conditions. |
| Blinding | This is not relevant to the study. The optical measurements are recorded automatically and cannot be biased by the investigator. |

# Reporting for specific materials, systems and methods

We require information from authors about some types of materials, experimental systems and methods used in many studies. Here, indicate whether each material, system or method listed is relevant to your study. If you are not sure if a list item applies to your research, read the appropriate section before selecting a response.

## Materials & experimental systems

| n/a | Involved in the study |
|---|---|
| ☒ | ☐ Antibodies |
| ☒ | ☐ Eukaryotic cell lines |
| ☒ | ☐ Palaeontology and archaeology |
| ☒ | ☐ Animals and other organisms |
| ☒ | ☐ Clinical data |
| ☒ | ☐ Dual use research of concern |

## Methods

| n/a | Involved in the study |
|---|---|
| ☒ | ☐ ChIP-seq |
| ☒ | ☐ Flow cytometry |
| ☒ | ☐ MRI-based neuroimaging |
